# Association of Sickle Cell Disease, Malaria and HIV in Multi Drug Resistant Invasive Non-typhoidal *Salmonella* Isolated from Outpatient and Hospitalised Children Below 16 Years in Informal Settlements in Nairobi County, Kenya

**DOI:** 10.24248/easci.v6i1.92

**Published:** 2024

**Authors:** Susan Mutile Kavai, Cecilia Mbae, Celestine Wairimu, Ronald Ngetich, Zillah Wakio, Robert Onsaŕe, Samuel Kariuki

**Affiliations:** a Centre for Microbiology Research, Kenya Medical Research Institute, Nairobi, Kenya; b Wellcome Sanger Institute, Hinxton, Cambridge CA10 1SA, United Kingdom; c Drugs for Neglected Diseases Initiative

## Abstract

**Background:**

Invasive non-typhoidal *Salmonella* (iNTS) disease continues to be a major public health problem, especially in sub-Saharan Africa (SSA), where incidence rates are 227 cases [range 152–341] per 100,000 populations. Populations at risk of iNTS include adults with human immunodeficiency virus (HIV) infection, malnourished children, and those with recent malaria or sickle-cell anaemia (SCA). In Kenya, iNTS disease is particularly a major challenge in poor informal settlements, with infants and young children less than 5 years of age being the most affected. Our study aimed to investigate the association between sickle cell disease, malaria, and HIV in multi-drug-resistant invasive non-typhoidal *Salmonella* from outpatient and hospitalised children ≤16 years in informal settlements in Nairobi County, Kenya.

**Methods:**

This study recruited 16,679 children aged ≤16 years who presented with salmonellosis symptoms for a period of 6 years (2013–2018). The patients were age-matched with controls (asymptomatic individuals). The study was conducted at 3 outpatient sites and 1 inpatient site; the outpatient sites were all located within the Mukuru informal settlement. The inpatient site was Mbagathi district hospital, which serves patients residing in Kibera informal settlement. Blood and stool samples from children with fever ≥38°C and/or diarrhea and stool samples alone from controls were collected for processing for the presence of iNTS using basic microbiology procedures including culture, serology, and Kirby Bauer disc diffusion for sensitivity testing. Dry blood spots were also taken and processed for sickle cell protein markers using high-performance liquid chromatography (HPLC). HIV and malaria tests were also conducted using rapid tests, respectively.

**Results:**

From the total of 22,246 blood and stool samples tested, 741 (3.3%) tested positive for *Salmonella* species. A total of 338 (45.6%) iNTS were isolated across all 4 sites; these consisted of 158 (21.3%) *Salmonella* Enteritidis and 180 (24.3%) *Salmonella* Typhimurium. The most common resistance phenotype was against ampicillin, chloramphenicol, and sulfamethoxazole trimethoprim. A total of 118 (34.9%) isolates were multidrug-resistant (MDR). Out of 2,684 dry blood samples subjected to HPLC for investigation of sickle cell disease traits, 1820 (67.8%) had normal haemoglobin (Hb AA/Hb AF); 162/2684 (6%) tested positive for sickle cell traits (Hb AS/Hb AFS). Some patients positive for iNTS were also found to have other co-morbidities; 4 (0.1%) tested positive for sickle cell disease (Hb FS), malaria, and HIV 8 (2.4%) and 5 (1.5%), respectively.

**Conclusion:**

The high prevalence of MDR iNTS isolates and emerging resistance to third-generation cephalosporins is of great concern, as they are the recommended drugs for the management of iNTS in our settings. Sickle cell disease, malaria, and HIV were all not major factors associated with iNTS disease among children in Mukuru and Kibera informal settlements.

## BACKGROUND

Globally, non-typhoidal *Salmonella* (NTS) disease is associated with an estimated 3.4 (range 2.1–6.5) million cases annually (overall incidence: 49 cases [range 30–94] per 100,000 population). Sub-Saharan Africa bears the highest burden with incidence (227 cases [range 152–341] per 100,000 population) and number of cases (1.9 [range 1.3–2.9] million cases); infants, young children, and young adults were most affected, with a case fatality rate between 20 and 25%.^1,2,3^ In SSA, iNTS disease is caused primarily by two serovars, *Salmonella* serovars Typhimurium and Enteritidis. It is a major challenge in SSA as it is responsible for increased cases of childhood morbidity and mortality.

Serovar *Salmonella* Typhimurium is more common than *Salmonella* Enteritidis.^4,5,6^ In most SSA countries, empirical treatment is often administered due to a lack of strengthened laboratory systems in the health care facilities.^7,8^ This greatly contributes to the inability to detect antimicrobial resistance, which can persist in the patient’s body system for many months.^9,10^

Invasive NTS disease (iNTS) in children in SSA has been associated with malaria infection, malnourishment, and sickle cell disease.^11,12^ Besides iNTS infections, patients with sickle cell disease (SCD) are susceptible to a variety of other bacterial infections, which are a major cause of morbidity and mortality.^13^ This increased susceptibility to infections is related to abnormalities in the defence mechanisms of these patients, including functional hyposplenism, an abnormality in the alternative pathway of complement activities, and defective neutrophil function.^13,14^ Devitalisation of the gut and bone due to repetitive vaso-occlusive crises, macrophage saturation with red cell breakdown products as a result of chronic haemolysis, and underlying splenic and hepatic dysfunction are known to predispose patients with SCD to salmonellosis.^13^ A reduction in iNTS disease incidence has been reported following an improvement in effective malaria control measures.^15^

Increasing antimicrobial resistance (AMR) in iNTS is of great global concern, and the situation is even more serious in low- and middle-income countries where empiric treatment options for effective treatment of life-threatening invasive disease are limited. Several studies have shown that strains of NTS that are multidrug resistant to recommended first-line antibiotics, including ampicillin, trimethoprim-sulfamethoxazole, chloramphenicol, and kanamycin, have emerged in several African countries over the past 20 years. Multi-drug resistance in iNTS has previously been reported in Kenya and Malawi,^4,10,16^ posing a major challenge to treatment and management options.^16^ *Salmonella* Typhimurium, sequence type 313 (ST313) is a distinct phylogenetic lineage that has emerged in Africa. It is now a significant cause of iNTS disease in Africa. A recent study reported isolating ST313 with both lineages I and II but fewer ST19 strains, an important cause of iNTS disease as well as asymptomatic carriage.^18^ Treatment failure and complications are associated with a lack of proper diagnostic capacity that can aid in the management of these multi-drug-resistant strains.^15,16,18^ We report on the association of sickle cell disease, malaria, and HIV in multi-drug-resistant invasive non-typhoidal *Salmonella* from outpatient and hospitalised children below 16 years in informal settlements in Nairobi County, Kenya.

## METHODS

### Study Site

Patients attending three outpatient facilities and one inpatient facility in Nairobi County were recruited for this study. The three outpatient facilities included the Medical Missionaries of Mary Clinic (MMM), Municipal County Council Clinic (MCC), and Mukuru kwa Reuben Clinic (MR). The outpatient site was from Mbagathi district hospital (MB) which is a level 4 referral facility. The participants recruited from Mbagathi district hospital were from Kibera informal settlement. Mukuru informal settlement is located 20km east of Nairobi city. It has a population of over 700,000 people while Kibera is located 6.6 kilometres from the city Centre with a population of over 2.5 million. The two informal settlements have similar characteristics of overcrowding, access to unlimited street foods, poor sewage infrastructure systems, flying toilets , and lack of clean drinking water.^8^

### Study Design

The study utilised a case control study design.

### Study Population and inclusion criteria

The study recruited 16,679 children aged 16 and under residing in Mukuru or Kibera informal settlements. Patients were included in the study if they presented with a history of fever lasting ≥ 3 days (an axillary temperature of ≥ 37.5 °C), reported having had ≥ 3 loose or liquid stools, and had not taken antibiotics at the time of presentation to the clinic sites. The study also recruited at least two age-matched controls for every case. These were children who presented to the clinic to attend the mother-child clinic for vaccination or came to the facilities presenting with non-typhoidal *Salmonella*-related symptoms.

### Exclusion Criteria:

Participants were excluded from the study if they were >16 years old, presented with non-typhoid-related symptoms, and had taken antibiotics at the time of presentation to the clinic sites.

### Sample Size Determination

The average NTS isolation rate from blood cultures in a previous investigation^7^ of NTS bacteremia in Nairobi was 4%; half of these isolates were also found in the faeces of the same patients. Using the Fishers technique^19^, a sample size (N) was determined by taking this as the working prevalence rate (P) for NTS from blood cultures at each study location, assuming an absolute precision (d) of 5% and a standard error (Z) from the mean of 1.96. Z²1-a P (1-P)

$$\text{N}=\frac{\;}{{\text{d}}^{2}}=\underset{0.0025}{1.96^{2}\text{X0}.06X0.94}$$

N=226Salmonellaisolates

Thus, a minimum of ~226 *Salmonella* isolates were required from blood, and 113 (half of 226) may be obtained from the stools of the same patients across all sites.

### Sample Collection and Transportation

Stool samples were collected from suspected iNTS cases as well as health-related individuals who presented to the clinics. A rectal swab was collected from participants who were unable to provide a stool sample. Rectal and stool swabs were placed in Cary Blair media (Oxoid, Basingstoke, UK) and transported under chilled conditions. For blood samples, 1 to 3 ml for < 5 years and 5 to 10 ml for 5 to 16 years, respectively, were collected from cases in a syringe, placed into blood culture bottles, and transported to a KEMRI laboratory.

### Sample Processing Methods Bacterial Isolation and Identification in Blood Samples

Blood cultures were incubated at 37°C in a BACTEC^™^ 9050 Blood Culture System (BD, Franklin Lakes, New Jersey, USA). The bactec flagged any positive blood cultures, and they were subsequently subcultured onto blood, chocolate, and MacConkey agar plates (Oxoid, Basingstoke, UK). The use of multiple media was to increase the chances of isolating iNTS. All blood cultures not flagged after 7 days were all removed from the bactec and subcultured to check for evidence of growth, then discarded. Suspect colonies from the primary cultures (pale colonies on MacConkey agar) were subjected to biochemical tests on API20E strips (API System, Montalieu Vercieu, France) and further typed by polyvalent O and monovalent antisera for iNTS.^8^

### Bacterial Isolation and Identification in Stool Samples

Stool and rectal swabs were placed into Selenite faecal broth media (Oxoid, Basingstoke, UK) for enrichment, and incubated at 37 °C for 18–24 hours. Bacterial growth from the Selenite faecal broth media was plated onto MacConkeyagar and Xyloselysinedeoxycholateagar (both from Oxoid, Basingstoke, UK). Non-lactose fermenters isolated from MacConkey agar were considered suspect colonies. These colonies were biochemically confirmed using the API20E system (Montalieu Vercieu, France). Serological confirmation of *Salmonella* sp. was done using polyvalent O and monovalent antisera for iNTS (Remel Europe Ltd.).

### Malaria Test (Rapid Detection Test)

Using a sterile lancet, a gentle prick was made towards the pulp of the 4th finger at the disinfected site. The first drop of blood was expressed by applying gentle pressure to the finger and wiped away with a dry piece of cotton wool. Gentle pressure was applied to the same finger until a new blood drop appeared. Using the blood collection device provided in the RDT kit (Biogenix, India) , the open end was gently immersed in the blood drop. The required volume of blood was collected as per the manufacturer’s instructions. The collected blood was transferred to the sample well. Holding the buffer bottle vertically, a drop of the buffer was added to the buffer well. Interpretation of the results was done according to the manufacturer’s instructions.

### Processing of Dried Blood Spots for Sickle Cell Diagnosis High Performance Liquid Chromatography (HPLC)

Using samples compared to a defined range of SC protein standards, the HPLC approach was used to diagnose SCD. Dry Blood Spots (DBS) were eluted, and the presence of normal and aberrant haemoglobins was determined by isoelectric focusing. Isoelectric focusing, which can detect sickle and normal haemoglobin with ease. On an HPLC screen, the gel bands were visually shown and independently compared to a standard control.^14^

### Antibiotic Susceptibility Testing

Invasive nontyphoidal *Salmonella* isolates were tested for susceptibility to 13 antibiotics using the Kirby-Bauer disc diffusion technique. These included: amoxicillin-clavulanic acid (AMC, 30 µg); ampicillin (AMP, 10 µg); cefotaxime (CTX, 30 µg); cefpodoxime (CPD, 10 µg); ceftazidime (CAZ, 30 µg), ceftriaxone (CRO, 30 µg); chloramphenicol (CHL, 30 µg); ciprofloxacin (CIP, 5 µg); gentamicin (GEN, 10 µg), kanamycin (KAN) (30 µg), nalidixic acid (NAL, 30 µg), sulfamethoxazole-trimethoprim (SXT, 25 µg), tetracycline (TET, 30 µg), (all from Oxoid Basingstoke, UK). A positive quality control organism; ATCC *Escherichia coli* was used. So in short remove the other organism, ATCC *Escherichia coli* 25922 and ATCC *Staphylococcus aureus* 25923, were used. The results were interpreted following the Clinical and Laboratory Standards Institute guidelines. MDR prevalence was defined as resistance to ampicillin, chloramphenicol, and sulfamethoxazole trimethoprim.^8^

### Data Analysis

Data from this study was analysed using the statistical package for social sciences (SPSS) version 20. Descriptive analysis was used to show the occurrence of NTS and social demographic factors such as age and gender. Antimicrobial-resistant patterns were interpreted and analysed using the M 100 Clinical and Laboratory Standards Institute (CLSI 2021). Antimicrobial-resistant patterns were analysed using WHONET 2021. https://whonet.org/.

### Ethical Considerations

The study was approved by the Scientific and Ethics Review Unit (SERU) of KEMRI (SSC No. 2076). All parents or guardians of participating children were informed of the study objectives, and voluntary written consent was sought and obtained before inclusion in the study. Participants aged 13–16 years, in addition to the written consent from their parents or guardians, also assented to participate in the study. Unique study and lab numbers deidentifying all study participants were used for the study. All patient documents, such as signed consents and data entry books, were stored in locked cabinets with limited access to the study staff. All patient data was stored on password-protected computers at KEMRI with authorised access only to the study team.

## RESULTS

Out of 22,246 patient samples collected, 741 (3.3%) were positive for *Salmonella* sp. From the 741 *Salmonella* sp, 338 (45.6%) were iNTS, 220 (65.1%) were cases (symptomatic), and 118 (34.9%) were controls (asymptomatic). The iNTS-positive patients with malaria were 8 (2.4%), 6 (1.8%) were cases, and 2 (0.6%) were controls, respectively. The iNTS-positive patients with HIV were 5 (1.5%) and were all cases.

### Antibiotic resistance among *S*. Typhimurium and *S*. Enteritidis (2013–2018)

**Figure 1:**
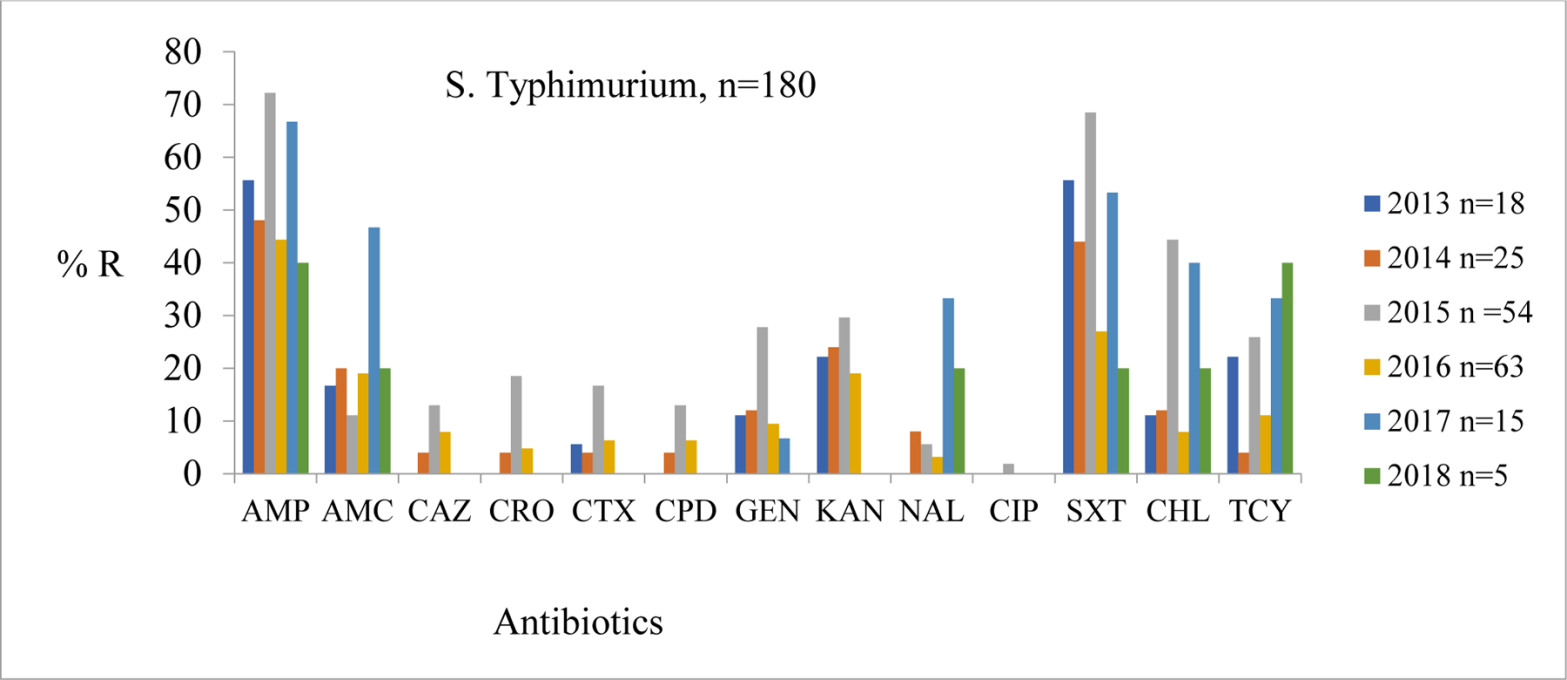
Comparison of Proportions of *S*. Typhimurium resistance profiles from cases and controls living Mukuru and Kibera informal settlements, Nairobi-Kenya Key: Ampicillin (AMP), Amoxicillin Clavulanate (AMC), Ceftazidime (CAZ), Ceftriaxone (CRO), Cefotaxime (CTX), Cefpodoxime (CPD), Gentamicin (CN), Kanamycin (K), Nalidixic Acid (NAL), Ciprofloxacin (CIP), Sulfamethoxazole-trimethroprim (SXT), Azithromycin (AZM), Chloramphenicol (C), and Tetracycline (TCY). No of isolates annually is represented by letter n.

The average resistance trends for *S.* Typhimurium (n = 180) against the first line antibiotics from 2013–2018 were as follows: ampicillin 98 (54.5%), sulfamethoxazole-trimethoprim 80 (44.7%), tetracycline 41 (22.8%), chloramphenicol 40 (22.6%). The resistance for the cephalosporins (Ceftazidime {CAZ}, ceftriaxone {CRO}, cefotaxime {CTX}, and cefpodoxime {CPD}) was generally low over the six-year period. In 2015, ampicillin and sulfamethoxazole-trimethoprim recorded the highest resistance at 38 (72.0%) and 37 (69.0%), respectively (Figure 1).

**Figure 2:**
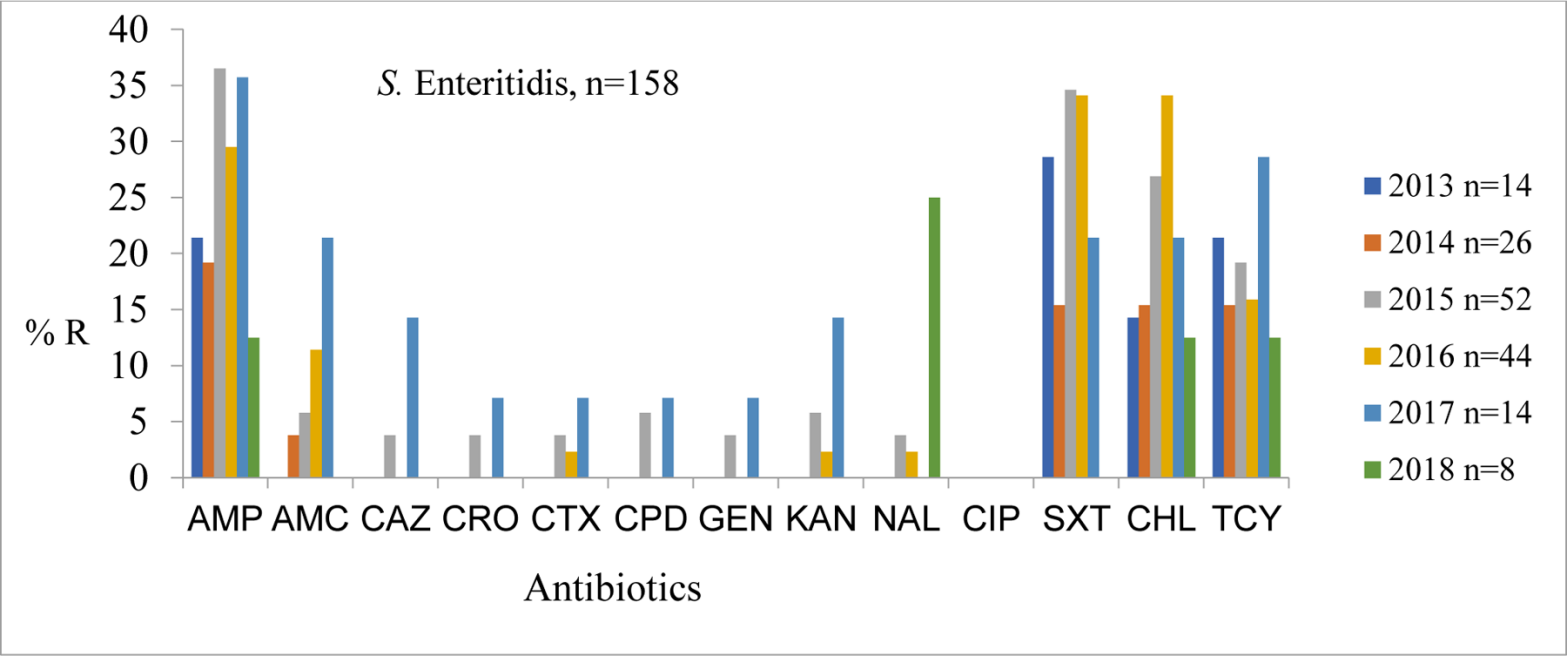
Comparison of Proportions of *S*. Enteritidis resistance profiles from cases and controls living Mukuru and Kibera informal settlements, Nairobi-Kenya Key: Ampicillin (AMP), Amoxicillin Clavulanate (AMC), Ceftazidime (CAZ), Ceftriaxone (CRO), Cefotaxime (CTX), Cefpodoxime (CPD), Gentamicin (CN), Kanamycin (K), Nalidixic Acid (NAL), Ciprofloxacin (CIP), Sulfamethoxazole-trimethroprim (SXT), Azithromycin (AZM), Chloramphenicol (C), and Tetracycline (TCY). No of isolates annually is represented by letter n.

Resistance trends for *S.* Enteritidis (n = 158) against first-line antibiotics were also quite high throughout the six-year period. Their average resistance was as follows: ampicillin 40 (25.8%), sulfamethoxazole-trimethoprim 35 (22.4%), chloramphenicol 32 (20.8%), and tetracycline 29 (18.83%). The resistance for the cephalosporins (Ceftazidime {CAZ}, ceftriaxone {CRO}, cefotaxime {CTX}, and cefpodoxime {CPD}) was generally low over the six-year period. In 2016, *S*. enteritidis had the highest resistance levels to sulfamethoxazole, trimethoprim, and chloramphenicol at 14 (34.0%) each (Figure 2).

### Occurrence of iNTS and social demographic factors

Age group 0–5 years had the most iNTS-isolated 215 (63.6%). Across all age groups, females were more affected than males, 185 (54.7%). It was also observed that stool samples had more iNTS isolated as compared to blood samples 216 (63.9%). There was a statistical significance of predisposition of children aged 0–5 and 11–16 years to *S*. Typhimurium; *P =*.001 and P =.002, respectively. There was also statistical significance in the predisposition of children aged 0–5 and 11–16 years to *S*. Enteritidis [*P =.0001* and P =.0005, respectively, at a confidence interval of 95%]

### Occurrence of iNTS and Sickle cell disease/Trait

A total of 2,684 samples from both blood and stool samples were subjected to HPLC for the processing of sickle cell disease. Out of these, 1820 (67.8%) tested normal haemoglobin, 162 (6.0%) were confirmed to have sickle cell trait, and 4 (0.2%) tested positive for sickle cell disease. Interestingly, 4 (0.2%) samples that tested positive for SCD tested negative for iNTS in both blood and stool samples.

### Occurrence of iNTS bacteraemia and Sickle cell disease, Malaria and HIV

A total of 47 iNTS were isolated from the blood samples subjected to the SCD test, and only 1 (2.1%) was positive for the sickle cell trait Hb AFS. 46 (97.9%) were found to have normal haemoglobin levels of 45 (Hb AA) and 1 (Hb AF). In addition, a total of 51 NTS were isolated from the stool samples, and 5 (9.8%) were positive for sickle cell trait 3 (Hb AFS) and 2 (Hb AS), while 46 (90.2%) were found to have normal haemoglobin 41 (Hb AA) and 5 (Hb AF). A total of 8 (2.4%) children positive for NTS were also positive for malaria but negative for HIV. These children came from a population of both cases and controls. *S*. Typhimurium was isolated in 7 malaria-positive patients, 5 cases, and 2 controls, while *S*. enteritidis was isolated in one malaria-positive patient. A total of 5 (1.5%) NTS-positive patients tested positive for HIV. Among these, 3 were cases, while 2 were controls (healthy individuals).

## DISCUSSION

Non typhoidal *Salmonella* (NTS) is one of the most prevalent pathogens in SSA that causes systemic infections in both adults and children. The NTS is made up of numerous serovars, the most frequently implicated pathogen being *S*. Typhimurium. Our study reported only two NTS serotypes, *S*. Typhimurium and *S*. Enteritidis, as the most common in Kenya. This is similar to other studies on iNTS disease in SSA previously described in Kenya^8^, Malawi ^18,^ Ghana^19^, DRC^20^ Burkina Faso^21^, and Mozambique.^22^

Our study showed the NTS isolates had high resistance levels against ampicillin, chloramphenicol, and sulfamethoxazole-trimethoprim. Emerging resistance to the third generation was also present. Another study conducted in Kenya revealed that NTS isolates from Kenya displayed resistance to ampicillin, chloramphenicol, sulfamethoxazole-trimethoprim, and cephalosporin’s more recently^23^. These two studies findings revealed that there is a possibility that resistance to cephalosporins could persist in the future. This is worrisome, as they are the recommended drugs for treatment of iNTS infections in Kenya. Another study conducted on MDR-NTS reported them as responsible for increased disease burden in SSA.^24^ This could be attributed to the ineffectiveness of first-line antibiotics due to their misuse over time.

Our study reported that the age group 0–5 years across all the sites had the highest number of iNTS infections. A similar study in Kenya^25^ revealed similar findings to our study: infants (below 1 year) were more likely to be infected with enteric pathogenic bacterial infection (OR 0.3, 95% CI 0.1–0.8) than the older ones. Another study done in Lusaka, Zambia, also implicated a high prevalence of infection among the ages of 0–12 months (61.3%).^26^ The high risk of iNTS and other enteric pathogen infections in these children could be due to their weak immune systems, which make them most susceptible to infections.

Our study did not report SCD, malaria, or HIV as comorbidities to iNTS. This was not in agreement with other studies reported in Kenya^27^, and sub-Saharan Africa.^28^ The difference in findings between our study and the others could be attributed to the low endemicity of malaria in Nairobi County, even though iNTS is endemic in urban informal settlements. A study reporting on invasive non-typhoidal Salmonella in sickle cell disease in Africa reported that NTS bacteremia overlaps significantly with malaria and HIV in Africa, both in terms of seasonality and affected age groups.^27,29^ Another study doing a literature review on the association between malaria and non-typhoidal *Salmonella* bacteremia in children in SSA demonstrated parallel decreases in the incidence of malaria and NTS bacteremia in the same geographical area over time.^28^ Our study showed a low co-occurrence of malaria, HIV, and SCD. This could be attributed to malaria not being endemic in the study area studied.

### Limitations

Our study recognises that it was conducted in an area that Malaria is not endemic hence it was not unusual that low levels of co-occurrence of Malaria, HIV, and SCD to iNTS were reported.

## CONCLUSION

Our study reports that first-line antibiotics such as ampicillin, chloramphenicol, and sulfamethoxazole trimethoprim may no longer be effective in the treatment of iNTS infections. However, there were low levels of resistance of NTS against third-generation cephalosporin hence they can still be used for treatment. Similarly, a vaccine targeting the under 5 years old children should be considered as a promising alternative to antibiotic treatment. Our study did not find a significant association between malaria, HIV, SCD, and iNTS due to the lack of endemicity of the comorbidities in the geographical area studied.
